# Pretreatment serum albumin as a predictor of cancer survival: A systematic review of the epidemiological literature

**DOI:** 10.1186/1475-2891-9-69

**Published:** 2010-12-22

**Authors:** Digant Gupta, Christopher G Lis

**Affiliations:** 1 Cancer Treatment Centers of America® at Midwestern Regional Medical Center, Zion, IL, USA

## Abstract

**Background:**

There are several methods of assessing nutritional status in cancer of which serum albumin is one of the most commonly used. In recent years, the role of malnutrition as a predictor of survival in cancer has received considerable attention. As a result, it is reasonable to investigate whether serum albumin has utility as a prognostic indicator of cancer survival in cancer. This review summarizes all available epidemiological literature on the association between pretreatment serum albumin levels and survival in different types of cancer.

**Methods:**

A systematic search of the literature using the MEDLINE database (January 1995 through June 2010) to identify epidemiologic studies on the relationship between serum albumin and cancer survival. To be included in the review, a study must have: been published in English, reported on data collected in humans with any type of cancer, had serum albumin as *one of the *or *only *predicting factor, had survival as one of the outcome measures (primary or secondary) and had any of the following study designs (case-control, cohort, cross-sectional, case-series prospective, retrospective, nested case-control, ecologic, clinical trial, meta-analysis).

**Results:**

Of the 29 studies reviewed on cancers of the gastrointestinal tract, all except three found higher serum albumin levels to be associated with better survival in multivariate analysis. Of the 10 studies reviewed on lung cancer, all excepting one found higher serum albumin levels to be associated with better survival. In 6 studies reviewed on female cancers and multiple cancers each, lower levels of serum albumin were associated with poor survival. Finally, in all 8 studies reviewed on patients with other cancer sites, lower levels of serum albumin were associated with poor survival.

**Conclusions:**

Pretreatment serum albumin levels provide useful prognostic significance in cancer. Accordingly, serum albumin level could be used in clinical trials to better define the baseline risk in cancer patients. A critical gap for demonstrating causality, however, is the absence of clinical trials demonstrating that raising albumin levels by means of intravenous infusion or by hyperalimentation decreases the excess risk of mortality in cancer.

## Introduction

Cancer is a major public health problem in the United States (US) and many other parts of the world. The World Health Organization (WHO) estimates that by 2020, globally, more than 15 million people will experience cancer and 10 million will die from it each year [1]. With the changing trends and advances in diagnostic aids, cancers can be diagnosed at much early age. Several important prognostic factors have been identified in the literature, some generic to all cancers and some specific for different cancer types. Some of the key factors determining cancer survival are age, stage [2,3], number of metastatic sites involved [4], location of metastases, tachycardia, blood counts [5,6], tumor markers [7,8], performance status (PS) [9,10], quality of life and malnutrition [11,12].

Malnutrition and cachexia in cancer patients are significant problems due to a variety of mechanisms involving the tumor, the host response to the tumor, and anticancer therapies [13]. Malnutrition has been associated with a number of clinical consequences, including deteriorated quality of life, decreased response to treatment, increased risk of chemotherapy-induced toxicity and a reduction in cancer survival [14]. There are various methods of assessing nutritional status in cancer, each with its own advantages and disadvantages [15]. Among the most commonly used tools to measure nutritional status are subjective global assessment (SGA) [16-18], bioelectrical impedance analysis (BIA) [19], and laboratory measurements of serum albumin [20], prealbumin, and transferrin [21,22]. Others include anthropometric parameters [21,23,24] such as weight loss, arm muscle circumference, skin-fold thickness [18,25], and presence of edema and ascites [26]. Though SGA is easy-to-use, inexpensive, and noninvasive, it is subjectively assessed and hence can be affected by inter-observer variation. Similarly, though BIA is easy-to-use, noninvasive, and reproducible, it relies on regression models derived in restricted samples of human subjects, which thus limits the usefulness of the derived model in other patients who differ from the original sample [27,28].

Serum albumin provides a simple method of estimating visceral protein function. Malnutrition and inflammation suppress albumin synthesis [29]. In an adult the normal range of serum albumin is defined as 3.5-5.0 g/dL and levels <3.5 g/dL is called hypoalbuminemia [2,3]. The inverse correlation between body weight index and albumin synthesis in cancer patients supports the possibility of a compensatory enhanced albumin synthesis in these metabolically affected patients. In the later stages of disease, malnutrition and inflammation suppress albumin synthesis [30]. As part of the systemic inflammatory response to the tumor, proinflammatory cytokines and growth factors are released [31,32] and have a profound catabolic effect on host metabolism. Interleukin-6, produced by the tumor or surrounding cells, stimulates liver production of acute-phase reaction proteins (such as C-reactive protein (CRP) and fibrinogen) in both the fasted and fed states. This increases the demand for certain amino acids, which if limited in the diet, may be obtained from breakdown of skeletal muscle. The lower serum albumin concentration may be due to the production of cytokines such as IL-6, which modulate the production of albumin by hepatocytes [33]. Alternatively, tumor necrosis factor may increase the permeability of the microvasculature, thus allowing an increased transcapillary passage of albumin. Presence of micrometastatic tumor cells in liver may induce the kupffer cells to produce a variety of cytokines (IL-Ib, IL-6 ve TNF), which may modulate albumin synthesis by hepatocytes [33,34]. Thus there is slight or no hypoalbuminemia in early stages of cancer but as the disease progresses albumin levels drop significantly and serve as good indicators of prognosis of cancer [33,34].

Serum albumin is generally used to assess the nutritional status, severity of disease, disease progression and prognosis. In the hospital setting, many reports have related serum albumin level to in-hospital mortality [35-38], length of stay (LOS) [39-41], and nosocomial infection [36,42]. Serum albumin has also been described as an independent prognosticator of survival in various cancers [43] like lung [12], pancreatic [6], gastric [44], colorectal [7,8,45] and breast [46]. Low serum albumin has also been shown to be an independent indicator for prognosis in cancer patients with unknown primaries [47]. However, these studies differ from each other with regard to population studied, study design, sample size, definition of low serum albumin used and factors adjusted for in the analyses. We therefore reviewed all available epidemiological literature (published within the last 15 years) to summarize the role of pretreatment serum albumin as an independent predictor of survival in cancer.

## Methods

We performed a systematic search of the literature using the MEDLINE database (January 1995 through June 2010) to identify epidemiologic studies on the relationship between serum albumin and cancer survival. We searched using the terms ''cancer survival or mortality or prognosis" in combination with the following terms: serum albumin, nutrition, serum proteins, predictors, and risk factors. We also searched the bibliography of the selected papers to identify relevant articles that we might have missed during the primary MEDLINE search. To be included in the review, a study must have: been published in English, reported on data collected in humans with any type of cancer, had serum albumin as *one of the *or *only *predicting factor, had survival as one of the outcome measures (primary or secondary) and had any of the following study designs (case-control, cohort, cross-sectional, case-series prospective, retrospective, nested case-control, ecologic, clinical trial, meta-analysis). There were no restrictions according to age, ethnicity or stage of cancer. As we were interested in empirical reports that have investigated the relationship between serum albumin and cancer survival, we did not include letters and meeting abstracts. All studies reviewed in this paper have been summarized in tables under separate headings by cancer type. This was primarily done to enable meaningful conclusions to be drawn separately for different cancer types as well as also to categorize studies into roughly equal groups. Within each table, studies were arranged chronologically by the year of publication starting with the most recently published study.

Although we did not formally rate the quality of reports, we recorded and present information on variables that may reflect the quality of reporting. These variables include study design, years of data collection, sample size, serum albumin cut-offs used, estimate of the association between serum albumin and cancer survival and inclusion of important prognostic factors in multivariate analyses.

## Results

The MEDLINE search identified 735 studies, which were assessed for relevance. Of these 735 studies, 175 studies were selected, and their abstracts were assessed for inclusion criteria by the authors. This exercise left 59 studies for the purpose of final inclusion and review in this manuscript.

### Gastrointestinal Cancer

Table 1 describes studies investigating the relationship between serum albumin and cancer survival in gastrointestinal cancer. The studies are arranged chronologically by the year of publication.

**Table 1:** Serum albumin and survival - gastrointestinal cancer

First author, year of publication, place	Year of data collection	Study design, Sample size	Cancer type	Groups being compared	RR (95%Cl), p-value	Variables adjusted for
Ishizuka M, 2009, Japan [48]	April 2005 to July 2007	Retrospective, 112	Colorectal	<3.5 g/dL >=3.5 g/dL	Univariate: 1.37 (1.10-1.71), 0.004 Multivariate: 2.38 (0.73-7.78), 0.14	Age, sex, tumor site, aspartate transaminase (AST), alanine transaminase (ALT), WBC, neutrophil, CA 19-9, CA 72-4, CRP

Neal CP, 2009, UK [49]	January 2000 to December 2005	Retrospective, 174	Colorectal	<4 g/dL >=4 g/dL	Univariate: 1.98 (1.21-3.25) 0.007 Multivariate: 1.68 (1.01-2.79) 0.04	Age, sex, site, stage, CEA, liver mets, chemotherapy, hematological indices, clinical risk score, Carstairs deprivation index

Sun LC, 2009, Taiwan [50]	January 1996 to December 2006	Retrospective cohort, 1367	Colorectal	<3.5 g/dL >=3.5 g/dL	Univariate: 1.72(1.38-2.14) < 0.001 Multivariate: 1.45(1.09-1.92) 0.011	Age, sex, site, tumor size, BMI, histology, UICC stage, CEA

Wang CY, 2009, Taiwan [51]	November 2002 to July 2007	Prospective, 123	Esophageal	>=3.5 g/dL <3.5 g/dL Also used as continuous variable	Univariate: p < 0.001 Multivariate: Categorical = 3.9, (1.9-8.2), <0.001 Continuous = 0.38, (0.25-0.58), <0.001	Age, histology, tumor location, stage, Serum CRP, BMI, WBC count, platelet, bilirubin, hemoglobin, BMI, treatment modality

Choi GH, 2008, South Korea [52]	March 1998 to January 2005	Retrospective, 97	Hepatocellular	<3.5 g/dL >=3.5 g/dL	Multivariate: 4.59 (1.79-11.75), 0.001	Sex, cirrhosis, AFP, platelets, tumor size, number of tumors, intrahepatic mets, venous invasion, Child-Pugh class, time to recurrence

Takahashi S, 2008, Japan [53]	March 1999 to September 2004	Retrospective cohort, 179	Hepatocellular	<3.5 g/dL >=3.5 g/dL	Univariate: p = 0.001 Multivariate: 3.75(1.64-8.56) 0.002	Age, sex, bilirubin, platelets, AFP, PIVKA-II, tumor size, tumor nodules

Di Fiore FD, 2007, France [2]	January 1997 to December 2003	Retrospective, consecutive case series, 105	Non-metastatic esophageal	<=3.5 g/dL >3.5 g/dL	Univariate: p = 0.007 Multivariate: 0.99 (0.50-1.98), 0.99	Age, sex, performance status, weight loss, BMI, hemoglobin, tumor location, tumor length, stage of disease, radiotherapy dose

Ishizuka M, 2007, Japan [3]	January 2001 to March 2006	Retrospective, 315	Colorectal	<=3.5 g/dL >3.5 g/dL	Univariate: 0.85 (0.53-1.343), 0.488 Multivariate: 1.98 (0.91-4.29), 0.082	Age, sex, tumor site, CEA, CA19-9, CA72-4, CRP, GPS

Siddiqui A, 2007, USA [6]	July 1986 to December 2004	Retrospective, 69	Pancreas	>=3.5 g/dL <3.5 g/dL	Univariate: p < .0001 Multivariate: 2.98 (2.20 to 3.76), <.0001	CA19-9, WBC, laboratory indicators, co-morbidities, age, sex, BMI, stage, treatment

Onate-Ocana LF, 2007, Mexico [44]	January 1987 to December 2002	Retrospective, 1023	Gastric	High: >=3.77 g/dL Medium: 3.3 to 3.73 g/dL Low: 2.81 to 3.29 g/dL Very low: <=2.3 g/dL	Medium: 1.2 (0.8-1.7), 0.31 Low: 1.2 (0.8-1.8), 0.31 Very low: 1.8 (1.3-2.6), 0.001	Stage of disease, lymph node dissection, gender, surgical resection

McMillan DC, 2007, UK [54]	January 1997 to June 2004	Retrospective, 316	Colorectal	<=3.5 g/dL >3.5 g/dL	GPS based on CRP and albumin was associated with survival (p < 0.0001)	Age, Sex, stage, adjuvant therapy

Nagaoka S, 2007, Japan [55]	January 1997 to November 1998	Cohort, 90	Hepatocellular	<3.5 g/dL >=3.5 g/dL	Univariate: 1.75 (1.13-2.70) 0.011 Multivariate: 2.01 (1.20-3.37) 0.008	Age, sex, hepatitis B virus, bilirubin, prothrombin time, platelet count, CRP, AFP, stage, initial treatment, AST, ALT

Read JA, 2006, Australia [56]	NA	Prospective consecutive case series, 51	Colorectal	<3.5 g/dL >=3.5 g/dL	Univariate: p = 0.017 Median survival in months >=3.5 g/dL = 14.3 <3.5 g/dL = 10.3	Gender, age, extent of prior therapy, extent of disease, PS, liver function CRP, PG-SGA, GPS, type of treatment

Liu SA, 2006, Taiwan [57]	March 1995 to December 2002	Retrospective, 1010	Oral	>=4.15 g/dL <4.15 g/dL	Multivariate: 1.313, (1.052-1.638), 0.016	Age, sex, complications, BMI, stage, recurrence/metastasis

Boonpipattanapong, T, 2006, Thailand [7]	October 1, 1998 to October 31, 2002	Retrospective cohort, 172	Colorectal	<3.5 g/dL >=3.5 g/dL	5-year survival <3.5 g/dL = 48% >=3.5 g/dL = 59%	CEA, tumor differentiation

Cengiz O, 2006, Turkey and USA [8]	1994-2003	Retrospective, 99	Colorectal	<=3.5 g/dL >3.5 g/dL	Univariate: <0.0001 Multivariate: 2.791, (1.37-5.67), 0.005	Age, gender, location, differentiation, hemoglobin, cholesterol, TNM stage, venous invasion, CEA, metastasis

Alici S, 2006, Turkey [58]	September 1999 to April 2002	Retrospective, 138	Gastric	<3 g/dL >=3 g/dL	Univariate: Median survival in years <3 g/dL: 1.7 >=3 g/dL: 8.8 p = 0.006	BMI, clinical stage, surgery, type of surgery, gender, age, PS, tumor grade, tumor location, hemoglobin. LDH, type of surgery

Arimura E, 2005, Japan [59]	January 1988 to December 2002	Prospective consecutive case series, 140	Hepatocellular	<=3.5 g/dL >3.5 g/dL	Univariate: 1.69 (1.01-2.84), 0.04 Multivariate: 1.49 (0.76-2.90), 0.24	LFTs, tumor size, tumor number, local recurrence, distant recurrence, AFP, ICG-R15 (%)

Schindl M, 2005, UK [60]	October 1, 1988, to January 31, 2002	Retrospective, 337	Colorectal	Continuous variable	Univariate: p < 0.001 Multivariate: 0.9 (0.9-1.0) p < 0.001	Dukes stage, site of primary tumor, diameter of the largest liver lesion, serum CEA, ALP, number of lesions, bilobar disease, age

Tateishi R, 2005, Japan [61]	January 1990 to December 1997	Prospective consecutive case series, 403	Hepatocellular	>3.5 g/dL (reference) 2.8-3.5 g/dL <2.8 g/dL	Univariate: 1.99 (1.52-2.59),0.0001 3.13 (2.01-4.88),0.0001 Multivariate: 1.74 (1.31-2.30) 0.00014 2.45 (1.55-3.88) 0.00013	Age, sex, treatment modality, tumor factors, including size, number of nodules, lobar distribution, and presence of extrahepatic metastasis, clinical manifestations, including ascites and hepatic encephalopathy, bilirubin, prothrombin activity, AST, ALT, platelet count, AFP, positivity for viral markers (hepatitis B surface antigen and anti-hepatitis C antibody), alcohol

Xu HX, 2005, China [62]	August 1997 to September 2003	Prospective consecutive case series, 137	Hepatocellular	<3.5 g/dL >=3.5 g/dL	Multivariate: 0.48 (0.28-0.83), 0.008	Age, gender, cirrhosis, Child's class, AFP, ALT, bilirubin, prothrombin time, tumor nodules, tumor size, treatment method, recurrence

Lien YC, 2004, Taiwan [63]	1987 to 1997	Retrospective, 314	Gastric cardia	>3.5 g/dL <=3.5 g/dL	Univariate: 5 year survival rate >3.5 g/dL: 38.4% <=3.5 g/dL: 19.1%, p = <0.001	Age, sex, extent of resection, diet at presentation, depth of penetration, nodal involvement

Elahi MM, 2004, UK [64]	1988 to 1996	Retrospective, 165	Colorectal Gastric	<3.5 g/dL >=3.5 g/dL	Median survival in months <3.5 g/dL: 1.7(0.6-2.8), >=3.5 g/dL: 6.9 (4.7-9.0) p = <0.001	Age, sex, GPS, tumor type, CRP

Chen MF, 2003, Taiwan [65]	1986 to 1998	Retrospective, 254	Hepatocellular	<=3.5 g/dL >3.5 g/dL	Univariate: Median survival in months <=3.5 g/dL: 6.18 >3.5 g/dL: 12.3, p = 0.0037 Multivariate: Disease-free survival 2.17 (1.21-3.90) Overall survival 1.65 (1.005-2.73)	Age, sex, Hepatitis B antigen, Hepatitis C antibody, AFP, BUN, creatinine, ALP, AST, bilirubin, prothrombin time, extent of resection, blood loss, blood transfusion, tumor size, no of tumors, resection margin, operating time

Koike Y, 2003, Japan [66]	1987 to 1999	Retrospective consecutive case series, 952	Hepatocellular	NA	Univariate analysis indicated that the serum albumin level was associated with survival	Child classification, number of tumor foci, portal venous invasion-targeted irradiation, and percutaneous tumor ablation of the parenchymal main tumor

Dixon MR, 2003, USA [67]	1991-1999	Retrospective cohort, 105	Colorectal	Continuous variable	Univariate (Median Albumin) (IQR): Short survival <120 days : 2.5 (2.2-3.0) Long survival >120 days : 3.1 (2.6-3.5), 0.002	Age, ALP, AST, total bilirubin, CEA, ALT, prothrombin time, mean corpuscular volume, fibrinogen, hematocrit, creatinine

Heys SD, 1998, UK [45]	1972 to 1985	Retrospective case series, 431	Colorectal	Continuous variable	Univariate: <0.00005 Multivariate: 0.95 (0.93-0.98) < 0.0001	Duke's stage, age and tumor differentiation

Stuart KE, 1996, USA [68]	1986-1995	Retrospective, 314	Hepatocellular	Albumin cutoffs not provided	Univariate: Median survival in months Low albumin: 4 High Albumin: 15, p < 0.001 Multivariate: p < 0.001	Age, gender, cirrhosis, alcohol abuse, bilirubin, PVO and AFP

Onate-Ocana LF, 2007, Mexico [69]	NA	Retrospective cohort, 793	Gastric	<=3.5 g/dL >3.5 g/dL	Multivariate: 1.26 (1.03-1.5), <0.03	TNM stage, operative morbidity, type of lymphadenectomy, gastrectomy performed

A study evaluating the influence of the modified Glasgow Prognostic Score (GPS) for prognostication of patients undergoing chemotherapy for unresectable colorectal cancer found it to be a an independent predictor of survival [48]. A study conducted to determine the prognostic value of pre-operative systemic inflammatory biomarkers and socioeconomic deprivation in patients undergoing resection of colorectal liver metastases found poor clinical risk score (3-5), high neutrophil count (>6.0 × 10(9)/l) and low serum albumin (<4 g/dL) to be the only independent predictors [49]. A study conducted in patients with colorectal cancer undergoing surgical treatment identified low serum albumin level, advanced Union for International Cancer Control (UICC) stage, and high carcinoembryonic antigen (CEA) level to be independent prognostic factors of cancer-specific survival [50]. A study done to evaluate if CRP and serum albumin were survival predictors of esophageal cancer demonstrated that only serum CRP concentration and hypoalbuminemia were independent prognostic indicators of survival [51]. A study analyzed the prognostic factors for survival after recurrence in hepatocellular carcinoma (HCC) and found that early recurrence (< or =12 months), Child-Pugh class B or C at diagnosis of recurrence, and serum albumin level of < or =3.5 g/dL at diagnosis of recurrence were poor prognostic factors for survival [52]. A study assessing the prognostic predictors in patients with HCC after radiofrequency ablation found low serum albumin, a high level of prothrombin induced by vitamin K absence or antagonist II (PIVKA-II), and multiple nodules to be independently prognostic of survival [53]. A study assessing the impact of baseline nutritional status on treatment response and survival in nonmetastatic patients with a locally advanced esophageal cancer treated with definitive chemoradiotherapy (CRT) found that in multivariate analysis, serum albumin level >3.5 g/dL was the only independent predictive factor of complete response to CRT (*P *= 0.009). However, for survival, independent prognostic factors were body mass index (BMI) >18 kg/m2, dysphagia Atkinson score, dose of RT >50 Grays and complete response to CRT [2]. Another study investigating the significance of preoperative GPS, that includes only serum CRP and serum albumin for postoperative prognostication of patients with colorectal cancer found that upon multivariate analyses using factors such as age, sex, tumor site, serum CEA, CA19-9, CA72-4, CRP, albumin, and GPS revealed that GPS was associated with postoperative mortality [3]. A study determined clinical and laboratory predictors of mortality in pancreatic cancer and found that upon multivariate analysis low serum albumin and an increased white blood cell (WBC) count independently predicted survival of less than 6 months [6].

Another study conducted to define the prognostic role of serum albumin in gastric cancer found that categorized pre-therapeutic serum albumin groups (medium, low and very low albumin) presented median survival times of 1.44 years, 1.96 years and 2.62 years respectively while the group of high albumin presented a mean survival of 10.68 years (P < 0.001). Multivariate analysis indicated that TNM staging system, surgical resection, type of lymph node dissection, gender and serum albumin were significant prognostic factors [44]. A study found that a combination of an elevated CRP and hypoalbuminaemia (GPS) was significantly associated with overall and cancer specific survival in colorectal cancer [54]. A study investigating the relationship between the serum levels of high sensitivity CRP (H-CRP) and the prognosis of HCC patients found positive H-CRP, albumin, tumor stage and initial treatment to be significant independent determinants of poor prognosis [55]. Another study evaluated novel inflammatory and nutritional prognostic factors in patients with advanced colorectal cancer. Using univariate analysis, significantly worse survival was found for patients with poorer performance status, high GPS, low albumin, elevated serum alkaline phosphatase (ALP), patient-generated subjective global assessment (PGSGA) score of >9. Upon multivariate analysis, type of treatment, PS, GPS, and ALP remained significant predictors of survival [56]. A study investigating whether nutritional factors could predict survival in oral cancer found upon multivariate analysis that those with a preoperative BMI of <22.8 kg/m2 tended to have a higher probability of death. In addition, those with a preoperative serum albumin level of <4.15 g/dL were generally associated with a poorer prognosis [57].

A study in colorectal carcinoma patients found that a preoperative CEA level greater than or equal to 5 ng/mL and albumin level less than 3.5 g/dL predict a poor survival chance for colorectal carcinoma patients. [7]. Another study investigating if pretreatment serum albumin and cholesterol levels were prognostic factors in patients with colorectal carcinomas concluded that a preoperative low level of serum albumin can be an indicator for the malignant potential of the tumor and represents an unfavorable prognosis for patients with colorectal carcinoma [8]. A study evaluated the effects of clinicopathological parameters and treatment approaches on survival in gastric carcinoma. With single variable analysis, BMI, clinical stage, surgery, type of surgery, and serum albumin were significant prognostic factors related to overall median survival time while on multivariate analysis, no surgical treatment, palliative surgery (compared with radical surgery), and BMI below 20 kg/m2 were found to be the statistically significant poor prognostic factors related to survival in multiple variable analysis [58].

A study conducted in 140 previously untreated cases of HCC found the indocyanine green retention at 15 min (ICG) test, tumor size, tumor number, and local recurrence to be the significant prognostic factors of survival upon multivariate analysis [59]. Another study in 337 patients with colorectal cancer liver metastases found Dukes stage, number of metastases, and serum concentrations of CEA, ALP, and albumin to be independent predictors of survival [60]. A study conducted in 403 patients with HCC found serum albumin, bilirubin, size of the tumor, and number of tumor nodules to be independent predictors of survival [61]. A study to identify prognostic factors for long-term outcome for patients with HCC after percutaneous microwave or radiofrequency ablation found incomplete ablation, serum albumin level, serum alpha-fetoprotein (AFP) level and Child-Pugh classification to be independent predictors of survival [62]. A study evaluating serum albumin as a prognostic factor for patient survival in cancer of gastric cardia found that in each cancer stage, the 5-year survival rate of patients with normal serum albumin levels was better than that among those with hypoalbuminemia. By multivariate analysis, serum albumin level and the pathologic T, N statuses were independent factors correlated with prognosis [63]. A study was done to assess the value of combination of hypoalbuminemia and an elevated circulating concentration of CRP as a prognostic score in patients with advanced gastrointestinal cancer and found that a cumulative score based on these two parameters was a useful prognostic indicator [64]. Another study investigated the prognostic factors in HCC patients without cirrhosis who underwent hepatectomy. By Cox regression analysis, serum ALP, albumin, multiple tumor status, and blood urea nitrogen were shown to be independent prognostic factors for the 5-year disease-free survival rates while serum albumin, blood transfusion, resection margin, and multiple tumors were shown to be significant independent factors that influenced overall survival rates [65]. Another study was done to clarify the factors contributing to the survival of HCC patients with portal venous invasion. Univariate analysis indicated that the serum albumin level, Child classification, number of tumor foci, portal venous invasion-targeted irradiation, and percutaneous tumor ablation of the parenchymal main tumor were significant. Multivariate analysis showed that percutaneous tumor ablation was the most important factor contributing to a favorable prognosis followed by number of tumor foci [66].

Another study found that patients with stage IV colon and rectal cancer with a CEA level greater than or equal to 275 ng/mL and an albumin level less than 2.7 g/dL had a significantly shorter survival time. Conversely, patients with an albumin level greater than or equal to 2.7 g/dL and a CEA level less than 275 ng/mL had a longer survival time [67]. A study investigated the prognostic value of serum albumin in colorectal cancer patients and found serum albumin, age, tumor stage (Dukes' stage) and tumor differentiation to be independent prognostic factors for survival [45]. Another study investigated prognostic factors at presentation in patients with HCC. Univariate analysis demonstrated that serum albumin, cirrhosis, AFP, and portal vein obstruction (PVO) were prognostic factors of high statistical significance. Multiple regression analysis yielded albumin, AFP, and PVO as the most powerful independent negative predictors of ultimate survival [68]. A study conducted in 793 patients with gastric cancer found TNM stage, operative morbidity, serum albumin, age, type of lymphadenectomy and gastrectomy performed to be independent prognostic factors [69].

A great majority of the studies reported in this section were retrospective and conducted in patients with colorectal cancer. The studies reviewed above highlight the importance of pretreatment serum albumin as an independent predictor of survival in patients with gastrointestinal cancer.

### Lung Cancer

Table 2 describes studies investigating the relationship between serum albumin and cancer survival in lung cancer. The studies are arranged chronologically by the year of publication.

**Table 2:** Serum albumin and survival - lung cancer

First author, year of publication, place	Year of data collection	Study design, Sample size	Cancer type	Groups being compared	RR (95%Cl), p-value	Variables adjusted for
Win T, 2008, UK [70]	2 years up to December 2006	Prospective consecutive case series, 110	Non Small Cell Lung	Continuous variable	Univariate: 0.93 (0.88-0.98), 0.006	Female gender, age, pneumectomy, chronic obstructive pulmonary disease, Smoking, Diabetes, Coronary disease, BMI, global quality of life

Forrest LM, 2005, UK [71]	January 2002 to December 2003	Prospective, 101	Non Small Cell Lung	>=3.5 g/dL <3.5 g/dL	Median Survival (months) (95% CI) For >/=3.5 g/dL = 8.7 (6.9-10.5) For <3.5 g/dL = 1.2 (0.0-2.8), p = <0.01	Age, sex, stage, hemoglobin, WBC, CRP, PS, GPS, treatment

Maeda T, 2000, Japan [9]	1978-1992	Retrospective, 261	Non Small Cell Lung	<3.5 g/dL >=3.5 g/dL	Median survival in months <3.5 g/dL = 5.0 >=3.5 g/dL = 9.6; p = <0.001 Multivariate: 1.69 (1.19-2.41), 0.0037	Age, gender, histology, PS, LFTs, Stage IV, bilirubin, CEA, liver metastases

Tas F, 1999, Turkey [10]	1991 to 1997	Retrospective, 207	Small Cell Lung	Normal: >=3.5 g/dL Low: <3.5 g/dL	Univariate: p = <0.001 Multivariate: p = 0.03	Age, gender, performance status, weight loss, clinical stage, hemoglobin, LDH, response to chemotherapy

Ray P, 1998, France [4]	NA	Retrospective, 99 patients with SCLC and 202 patients with NSCLC	Small cell lung and Non-Small cell lung	NA	Serum albumin levels were not found to be associated with survival	Tumor, node, metastasis status, PS, body weight loss, WBC, serum sodium, LDH, ALP, serum NSE, serum TPS, and CYFRA 21-1

Lai SL, 1998, Taiwan [72]	NA	Prospective, 150	Non Small Cell Lung	NA	Patients who died within six months after diagnosis had significantly lower values of all nutritional parameters than those who survived more than 6 months	Weight/height ratio, percent of standard triceps skin-fold thickness, percent of standard arm muscle circumference, transferrin, creatinine height index and total lymphocyte count

Maestu I, 1997, Spain [73]	November 1981 to January 1993	Retrospective, 341	Small cell lung	<3.4 g/dL >=3.4 g/dL	Univariate: 0.0057 Multivariate : coefficient = -0.3457, p = 0.001	LDH, disease extent, CK, neutrophils, PS, glycemia, ESR, sodium, potassium, ALP, urea, uric acid, age

Muers MF, 1996, UK [74]	NA	Retrospective consecutive case series, 207	Non Small Cell Lung	NA	Prognostic Index = -0.42 × distant metastases + 1.1 × hoarseness + 0.47 × malaise - 0.34 × immediate treatment intent + 0.72 × lymphocyte count + 0.94 × serum albumin + 0.62 × sodium - 0.98 × ALP	Sex, the activity score, the presence of malaise, hoarseness and distant metastases at presentation, and lymphocyte count, sodium and ALP levels

Hespanhol V, 1995, Portugal [75]	1984 to 1990	Prospective, 411	Non Small Cell Lung	<3.5 g/dL >=3.5 g/dL	Univariate: 1.92, (1.55-2.36), 0.000 Multivariate: 0.588 (0.46-0.74), 0.000	PS, Weight loss, Hoarseness, stage, lymphocyte, LDH, sex

Espinosa E, 1995, Spain [76]	1980-1992	Retrospective onsecutive case series, 292	Non Small Cell Lung	>=4 g/dL <4 g/dL	Univariate: Median survival in months For >=4 g/dL = 9 For <4 g/dL = 7, p = 0.004; Multivariate: Coefficient = -2.52, p = 0.0001	Number of metastases, LDH, PS, sedimentation rate

A study was done to identify prognostic factors in patients with potentially curable lung cancer. Factors significantly (p < 0.05) associated with poor overall survival were age at assessment, diabetes, serum albumin, peak VO2 max, shuttle walk distance, and predicted postoperative transfer factor [70]. The value of an inflammation-based prognostic score (GPS) was compared with PS in a longitudinal study of patients with inoperable non small cell lung cancer (NSCLC). At diagnosis, stratified for treatment, only the GPS (Hazard Ratio (HR) 2.32, 95% Confidence Interval (CI) 1.52-3.54, P < 0.001) was a significant predictor of survival. In contrast, neither the GPS nor PS measured at 3-6 months follow-up were significant predictors of residual survival [71]. Another study analyzed prognostic factors in patients with advanced NSCLC who had been enrolled in clinical trials conducted by the Okayama Lung Cancer Study Group. PS, clinical stage, liver metastasis or serum albumin level was an independent prognostic factor by Cox's analysis [9]. A study was conducted to investigate the distribution of metastatic lesions and their influence on survival, as well as other prognostic factors on the outcome of patients with extensive small cell lung cancer (SCLC). Response to treatment was the most important prognostic factor; while clinical stage, weight loss, performance status, gender and serum lactate dehydrogenase (LDH) and albumin levels were other relevant parameters in predicting the outcome of patients with SCLC (p = 0.05) [10].

A study was done to determine predictive factors of treatment response and survival in SCLC and NSCLC. In SCLC, the significant determinants of poor survival were lack of complete response (HR: 2.04), weight loss (HR: 1.76), high serum LDH level (HR: 1.64), and high serum TPS level (HR: 2.47). In NSCLC, significant determinants of poor survival were no objective response (HR: 2.28), poor performance status (HR: 2.52), presence of metastases (HR: 1.51), and high serum CYFRA 21-1 level (HR: 1.84) [4]. A study conducted to assess the impact of nutritional status on survival in lung cancer found that patients who died within six months after diagnosis had significantly lower values of all nutritional parameters than those who survived more than six months. Patients with more abnormal parameters tended to have poorer survival rates [72]. Another retrospective analysis was done to identify which pretreatment clinical or blood parameters were predictive of patient survival in small-cell lung cancer (SCLC). Significant prognostic factors for survival after univariate and multiple regression analysis were: disease extent, PS, creatine kinase, neutrophilia, LDH, hypoalbuminemia, hyperglycemia and bicarbonate [73].

A group of consecutive patients with NSCLC was studied and the prediction of their physicians as to how long they would survive (in months) was compared with their actual survival. A prognostic index was also developed using features recorded at the patients' initial presentation. Using Cox's regression model, the sex of the patient, the activity score, the presence of malaise, hoarseness and distant metastases at presentation, and lymphocyte count, serum albumin, sodium and ALP levels were all identified as useful prognostic factors [74]. Another study assessed the influence on survival of 21 clinical, anatomical, hematological and biochemical factors in 411 patients with advanced NSCLC. The main determinants of survival were found to be performance status, weight loss and serum albumin. Other factors such as the staging (presence or absence of metastasis), lymphocytes, lactic dehydrogenase and hoarseness were also significant [75]. A study was done with an objective to find factors related to response, the duration of response and overall survival in patients with advanced NSCLC. The following factors were predictive for survival: weight loss, performance status, lymphocyte count, albumin level, number of metastases and the presence of bone metastases. It concluded that the albumin level identifies a group of patients with advanced NSCLC who are more likely to respond to cisplatin-containing chemotherapy [76].

Studies reported in this section were primarily conducted in patients with NSCLC and demonstrate the prognostic significance of pretreatment serum albumin in predicting patient survival.

### Female Cancers

Table 3 describes studies investigating the relationship between serum albumin and cancer survival in female cancers. The studies are arranged chronologically by the year of publication.

**Table 3:** Serum albumin and survival - female cancers

First author, year of publication, place	Year of data collection	Study design, Sample size	Cancer type	Groups being compared	RR (95%Cl), p-value	Variables adjusted for
Gupta D, 2009, USA [77]	January 2001 to May 2006	Retrospective, consecutive case series, 213	Ovarian	>=3.5 g/dL <3.5 g/dL Used as continuous as well	Univariate: median survival in months (95%CI) Low: 7.3 (4.8 to 9.8) Normal: 23.3 (16.5 to 30.1); p < 0.0001 Multivariate: 0.39 (0.29-0.53), <0.001	Age, BMI, CA125, tumor stage, treatment history

Sharma R, 2008, UK [78]	October 2003 to June 2006	Retrospective, 154	Ovarian	<3.5 g/dL >=3.5 g/dL	Univariate: 1.71 (0.92-3.18), 0.091 GPS score was prognostic on multivariate analysis	Tumor type, stage, grade, ascites, debulking surgery, ALP, residual disease, CRP

Alphs HH, 2006, USA [79]	January 1, 1990 to June 30, 2004	Retrospective, 78	Ovarian and primary peritoneal	>=3.7 g/dL <3.7 g/dL	Univariate: 0.58 (0.42-0.79), p < 0.00 Multivariate: 0.60 (0.41-0.89), p = 0.01	Age, race, BMI, Co-morbidity index, surgeon, ASA score, tumor size, intraoperative blood loss, ascites

Lis CG, 2003, USA [46]	March 1993 to December 1999	Retrospective consecutive case series, 180	Breast	>=3.5 g/dL <3.5 g/dL	Multivariate: 3.53, 0.0033	Abnormal breast antigen, tumor stage, abnormal HER2/Neu readings

Wyld L 2003, UK [80]	January 1997 to January 2002	Retrospective, 145	Breast cancer with liver metastases	First group <3.0 g/dL >=3.0 g/dL Second group <3.5 g/dL >=3.5 g/dL	Median survival in months (95%CI) For first group >=3.0 g/dL = 5.86 (0.16 - 51) <3.0 g/dL = 1.5 (0.16 - 5.13), p = 0.01 For Second group >=3.5 g/dL = 7.0 (0.27 - 51) <3.5 g/dL = 2.0 (0.16 - 27.2) p = 0.01	LFTs, CEA, bilirubin, age, histological grade, ER status, metastasis, treatment response

Clark TG, 2001, UK [81]	01/01/1984 to 31/12/1999	Retrospective, 1189	Ovarian	Continuous variable	Univariate: p <= 0.05 Multivariate: 0.97 (0.96, 0.99), 0.036	Age, FIGO stage, the presence or absence of ascites, performance status, histology, debulking, grade, CA125 and ALP

A study investigating the prognostic role of serum albumin in patients with ovarian cancer treated in an integrative cancer treatment setting found that every one gm/dL increase in serum albumin was associated with a RR of 0.39 (95% CI: 0.29 to 0.53; p < 0.001) [77]. A study investigated whether an inflammation-based prognostic score (GPS) was associated with survival in patients with advanced stage (stage III/IV) ovarian cancer. Patients with both an elevated CRP (>10 mg/l) and hypoalbuminaemia (<3.5 g/dL) were allocated a GPS score of 2. Patients in whom only one or none of these biochemical abnormalities was present were allocated a score of 1 or 0, respectively. On multivariate analysis, a high GPS score, non-serous histology, high ALP and no initial surgery were independent predictors of worse overall survival [78]. A study conducted to identify perioperative variables predicting surgical outcome and survival among elderly women diagnosed with ovarian and primary peritoneal cancer found that patients older than 80 years were associated with a nearly 2-fold increase risk of mortality while those with preoperative albumin levels ≥3.7 g/dL were associated with a 40% reduction in mortality risk [79]. A study investigated the effect of baseline serum albumin levels on 180 breast cancer patients. Univariate statistical analysis found that low levels of serum albumin adversely affected survival by a statistically significant level for all stages of breast cancer while Cox regression analysis found that normal levels of albumin (>3.5 g/dL) reduced the risk of death by 72% (*p *= .0033) [46]. Another study in patients of breast cancer with secondaries in liver found that factors that significantly predicted a poor prognosis on univariate analysis included symptomatic liver disease, deranged liver function tests (LFTs), the presence of ascites, histological grade 3 disease at primary presentation, advanced age, estrogen receptor (ER) negative tumors, CEA of over 1000 ng/ml and multiple vs single liver metastases. Multivariate analysis of pretreatment variables identified a low albumin, advanced age and ER negativity as independent predictors of poor survival [80]. Another study developed a prognostic model using Cox regression in 1189 primary cases of epithelial ovarian cancer and found that the significant (*P *≤ 0.05) prognostic factors for overall survival were age at diagnosis, FIGO stage, grade of tumor, histology (mixed mesodermal, clear cell and endometrioid versus serous papillary), the presence or absence of ascites, albumin, ALP, PS, and debulking of the tumor [81].

All studies reviewed in this section were retrospective and conducted primarily in patients with ovarian cancer. All studies found pretreatment serum albumin to be prognostic of cancer survival.

### Multiple Cancers

Table 4 describes studies investigating the relationship between serum albumin and cancer survival in multiple cancer sites together. The studies are arranged chronologically by the year of publication.

**Table 4:** Serum albumin and survival - multiple cancer sites

First author, year of publication, place	Year of data collection	Study design, Sample size	Cancer type	Groups being compared	RR (95%Cl), p-value	Variables adjusted for
Penel N, 2008, USA [82]	October 1997 to October 2002	Retrospective consecutive case series, 148	Breast, colon, rectum, head and neck, lung, others	>=3.8 g/dL <3.8 g/dL	Univariate: Median overall survival (days) <38 g/l: 91 (1-2421) >=38 g/l: 363 (296-429), p = 0.00001 Multivariate: 2.51 (1.51-4.18); 0.0001	Primary site, liver metastases, other viscera metastases, BMI, lymphocyte count, granulocyte count

Lam PT, 2007, Hongkong [12]	January to December 2002	Prospective cohort, 170	Lung, liver, lower gastrointestinal tract, breast, gynecological, haematological, nasopharyngeal, prostate, unknown, others	Continuous variable	Univariate: 0.94 (0.91-0.96), <0.001 Multivariate: 0.95 (0.92-0.98), 0.001	Demographic data, tumor characteristics, blood parameters, functional status, comorbidities, total symptom score, and psychosocial parameters

Santarpia L, 2006, Italy [83]	January 1996 to September 2003	Retrospective, 152	Stomach, ovaries, colorectal, endometrium, breast, ileum, gallbladder, pancreas, kidney, skin, prostate, abdominal sarcoma, unknown	Mean (SD) 2.8 +/-0.6 g/dL 3.1+/- 0.5 g/dL 3.3+/- 0.6 g/dL	Survival in days For 2.8 +/-0.6 g/dL = <30 days For 3.1+/- 0.5 g/dL = 30-90 days For 3.3+/- 0.6 g/dL = >90 days p = 0.001	Age, gender, height, weight, BMI, hemoglobin, lymphocyte count, cholesterol, CHE, KPS score, pain, ascites, vomiting

Pasanisi F, 2001, Italy [84]	1995-1999	Retrospective consecutive case series, 76	Stomach, colorectal, ovary, others	Mean (SD) 3.13 +/- .51 g/dL 3.57 +/- .43 g/dL	Survival in months For 3.13 +/- .51 g/dL <= 3 months For 3.57 +/- .43 g/dL > 3 months P = 0.002	Age, weight, BMI, hemoglobin, lymphocyte count, cholesterol, pain and ascites

Vigano A, 2000, Canada [85]	July 1, 1996, to December 31, 1998	A prospective cohort of 227 consecutive patients	Breast, gastrointestinal, lung	>=3.5 g/dL <3.5 g/dL	Univariate: 1.9 (1.4-2.8), <0.01 Multivariate: 7.3 (2.9-18.1)	Lung primary tumor, presence of liver metastases, tumor burden, co morbidity, performance status, weight loss, lymphocyte count, nausea, LDH

Maltoni M, 1997, Italy [86]	NA	Prospective consecutive case series, 519	Solid tumors excluding renal cancer and hematological cancer	Normal: 3.3-5.5 g/dL Low: 2.7-3.2 g/dL Very low: <=2.6 g/dL	Univariate: p = 0.0015 Median length of survival (days): Normal =40.0 low = 29.5 Very low = 24.0 months	Total WBC, neutrophil percentage, lymphocyte percentage, proteinuria, pseudocholinesterase

A study retrospectively assessed prognostic factors in cancer patients screened for Phase1 trials. The univariate analysis identified PS ≥ 1, BMI < 20 kg/m2, other primary sites (excluding breast, lung, head and neck and colon and rectum), presence of liver metastases, presence of other visceral metastases, serum albumin <38 g/l, lymphocyte count <700/mm3 and granulocyte count >7500/mm3 as poor prognostic factor for overall survival. The Cox model identified serum albumin and lymphocyte count as independent prognostic factors [82]. A study done to identify potential factors affecting survival in patients with advanced cancer in a local palliative care unit found age, number of involved metastatic sites, serum albumin, PS score, and Edmonton Symptom Assessment System score were independent prognosticators [12]. Another study done in patients with carcinomatosis on home parenteral nutrition found traditional parameters (PS, albumin, pain, and vomiting) and cholinesterase level to be useful survival predictors [83]. Clinical, anthropometric, hematologic, and biochemical variables, evaluated immediately before starting nutritional treatment, were related to survival in 76 terminal-cancer patients with irreversible bowel obstruction receiving home parenteral nutrition. With regard to bivariate and multivariate analyses, the linear correlation indicated that survival was associated with albumin (*r *= 0.489, *P *= 0.001) and hemoglobin (*r *= 0.300, *P *= 0.008) but not with age, weight, BMI, lymphocyte count, or cholesterol [84].

A study done to establish the predictors of survival in patients with terminal cancer found that shorter survival was independently associated with a primary tumor of the lung (vs breast and gastrointestinal tract combined), liver metastases, moderate to severe co morbidity levels (vs absent-to-mild levels), weight loss of greater than 8.1 kg in the previous 6 months, serum albumin levels of less than 3.5 g/dL, lymphocyte counts of less than 1 × 10^9^/L, serum LDH levels of greater than 618 U/L, and clinical estimation of survival by the treating physician of less than 2 months (vs 2-6 and >6 months) [85]. A multicenter study assessed the role of 13 hematological and urinary parameters in 530 terminally ill cancer patients. A poor prognosis was predicted by high total WBC count, high neutrophil percentage, low lymphocyte percentage, low serum albumin levels, low pseudocholinesterase levels, and high proteinuria [86].

Studies reviewed in this section were conducted in patients with a wide range of cancer types including breast, colon, head and neck, lung, liver and gynecological. All studies found pretreatment serum albumin to be prognostic of cancer survival.

### Other Cancer Sites

Table 5 describes studies investigating the relationship between serum albumin and cancer survival in other less common cancer sites. The studies are arranged chronologically by the year of publication.

**Table 5:** Serum albumin levels and survival - other cancer sites

First author, year of publication, place	Year of data collection	Study design, Sample size	Cancer type	Groups being compared	RR (95%Cl), p-value	Variables adjusted for
Barreto-Andrade JC, 2009, Mexico [87]	January 1986 to May 2006	Retrospective, 61	Soft Tissue Sarcoma	Low <3.5 g/dL Normal >=3.5 g/dL	Univariate: p = 0.03 Multivariate: p = 0.02	Age, sex, obesity, previous biopsy performed, histology, site histologic grade, stage, tumor resectability, tumor size, performance status, surgical risk

Seve P, 2006, France [47]	January 1, 1998 to December 31, 2004	Retrospective consecutive case series, 317	Unknown Primary	Low <3.5 g/dL Normal >=3.5 g/dL	Univariate: Median survival in days Low: 62; Normal: 318; p < 0.0001 Multivariate: 2.70 (1.79-4.07), <.0001	Age, sex, ACE-27 score, No. of sites, liver metastasis, peritoneal mets, PS, LDH, ALP, hemoglobin, platelets

Alici S, 2003, Turkey [88]	1989 to 1998	Retrospective, 110	Non-Hodgkin's Lymphoma	Normal Low	Univariate: p = 0.005 Multivariate: p = 0.022	Age, sex, stage, PS, B symptoms, treatment regimen, remission status, histology, bulky disease, LDH, ESR, extranodal involvement

Medow MA, 2002, USA [89]	July 1993 to June 1997	Retrospective consecutive case series, 406	Head and neck	<3.85 g/dL >=3.85 g/dL	TNM stage IV or recurrent disease Median survival: <3.85 g/dL: 404 days (286-532 days), >=3.85 g/dL: 625 days (536-1032 days)	Age, tumor stage, self-reported functional class, systolic blood pressure, diastolic blood pressure, and BMI

Ljungberg B, 2000, Sweden [90]	April 1982 to February 1999	Retrospective consecutive case series, 106	Renal cell	Continuous variable	Univariate: NA, p = 0.063 Multivariate: 1.01 (0.45 - 2.28), 0.96	Age, gender, tumor size, PS, solitary metastases, calcium, ESR, nuclear grade, DNA ploidy and vein invasion

Schwartzbaum JA, 1999, USA [91]	February 1, 1993 to December 31, 1995	A convenience sample, 24	Glioblastoma multiforme	1^st ^Quartile (2.6-3.1 g/dL) 2^nd ^Quartile 3^rd ^Quartile 4^th ^Quartile (3.9-4.4 g/dL)	Multivariate: 2^nd^= 1.2 3^rd^= 0.1 4^th ^=0.1 p = 0.007	Age, sex, chemotherapy, serum iron, radiation

Aparicio J, 1998, Spain [92]	1970 to 1993	Retrospective, 116	Ewing's sarcoma	Low: <=3.5 g/dL Normal: >3.5 g/dL	Univariate: 5 year survival 48% in normal and 7% in low; median survival 52 months in normal and 6 months in low, p < 0.0001 Multivariate: p = 0.001	Age, sex, tumor site, maximum tumor diameter, extent of disease, PS, duration of symptoms before diagnosis, systemic symptoms, leukocytes and hemoglobin, ESR, LDH, histologic pattern, percent of tumor necrosis on the initial biopsy specimen

Citterio G, 1997, Italy [93]	1988 onwards	Retrospective consecutive case series, 109	Renal cell	NA	Univariate: p < 0.01	Age, sex, DFI, PS, stage at diagnosis, grading, number and type of metastatic sites, nephrectomy, blood levels of hemoglobin, creatinine, calcium, LDH, ferritin, ALP, triglycerides

A study analyzing a group of 61 patients with soft tissue sarcomas found advanced stage, high tumor grade, irresecability, and serum albumin as independent prognostic factors of survival upon multivariate analysis [87]. A study investigated how lymphopenia and low serum albumin could predict prognosis of patients with carcinoma of unknown primary (CUP). The results from multivariate analysis showed that patients who had a PS >= 2 (using the World Health Organization scale), a high overall comorbidity score (on the Adult Comorbidity Evaluation 27), liver metastasis, elevated serum LDH levels, lymphopenia (defined as an absolute lymphocyte count >=0.7 × 10^9^/L), and low serum albumin levels had a worse prognosis. Lymphopenia and low serum albumin levels were identified as 2 new independent markers of prognosis in patients with CUP [47]. A study conducted to identify the prognostic factors that specifically predict survival of patients with localized aggressive Non Hodgkin's Lymphoma (NHL), found incomplete response, low serum albumin, bulky disease (>10 cm), and high grade histology to be independent predictors of survival [88]. In a study on head and neck cancer patients, age, TNM tumor stage, functional class, systolic and diastolic blood pressure, BMI, and serum albumin concentration were evaluated as predictors of survival. Patients with stage IV or recurrent squamous cell carcinoma could be stratified by either serum albumin concentration or by age into 2 groups with a median survival of 1 or 2 years [89]. In another study a number of variables were analyzed to identify factors that might predict the survival time in renal carcinoma. A number of factors correlated to survival time in univariate analysis, including solitary versus multiple metastases, serum albumin and DNA ploidy, but after Cox multivariate analysis their significance was lost [90]. To determine whether serum albumin levels, before first surgery, predict time until death, 24 glioblastoma multiforme patients were studied. Patients with presurgical serum albumin levels below 3.4 g/dL survived an average (median) of 62 days (95% confidence interval (CI): 34, 135 days) after surgery. Those with serum albumin levels of at least 3.4 g/dL survived an average of 494 days (95% CI: 241, 624 days). It was concluded that presurgical serum albumin levels can be used to evaluate the success of randomization of clinical trials for glioblastoma multiforme therapies [91]. Another showed that raised serum LDH levels, hypoalbuminemia and distant metastases at diagnosis were independent adverse prognostic factors in 116 patients with Ewing's sarcoma [92]. A study done with an objective of determining prognostic factors for survival in renal cancer patients found the following variables to be statistically significant at the univariate analysis (p < 0.01): disease free interval (DFI), PS, stage at diagnosis, grading, nephrectomy, sites of metastases, blood hemoglobin, serum albumin, calcium, LDH, ALP [93].

Most of the studies reviewed in this section were retrospective and conducted in a wide range of cancer types including renal, head and neck, glioblastoma multiforme, NHL, soft tissue sarcoma, Ewing's sarcoma and unknown. All studies found pretreatment serum albumin to be prognostic of cancer survival.

## Discussion

Ecological and observational studies suggest that low serum albumin is associated with higher mortality from cancer. Research conducted over the last decade or so has demonstrated that serum albumin levels (either considered alone or in combination with other parameters) can provide useful prognostic information in a variety of cancers. For example, some studies have used an inflammation based score, which is derived from the acute-phase proteins CRP and albumin and is termed the GPS. The GPS has been defined as follows: patients with both an elevated CRP (>10 mg/l) and hypoalbuminemia (<3.5 g/dL) are allocated a score of 2; patients in whom only one of these biochemical abnormalities is present are allocated a score of 1; patients in whom neither of these abnormalities is present are allocated a score of 0. With CRP > 10 mg/L and serum albumin levels >=3.5 g/dL the HR was 2 (CI = 1.47-2.70 and p < 0.001) [3,48,51,54]. In this paper, we systematically review all available epidemiologic literature on the relationship between pretreatment serum albumin and cancer mortality.

Of the 29 studies reviewed on cancers of the gastrointestinal tract, 23 studies were retrospective and 6 were prospective. Majority of the studies were conducted in colorectal and hepatocellular cancer. The sample size studied ranged from 51 to 1367. Serum albumin was either used as a categorical variable (with 3.5 g/dL as the most commonly used cut off) or continuous variable. Some studies used different cut offs such as 4 g/dL [49] and 4.15 g/dL [57]. Age, sex, white cell count, stage of the tumor, tumor site, PS, BMI and LFTs were the most commonly adjusted variables in the multivariate analysis. All except three studies [58,59,66] found higher serum albumin levels to be associated with better survival in multivariate analysis.

Of the 10 studies reviewed on lung cancer, 4 were prospective and 6 were retrospective. 7 studies were done in NSCLC patients, 2 in SCLC and 1 study included both NSCLC and SCLC patients. The sample size studied ranged from 101 to 411. Serum albumin was either used as a categorical variable (with 3.5 g/dL as the most commonly used cut off) or continuous variable. Some studies used different cut offs such as 3.4 g/dL [73] and 4 g/dL [76]. Age, sex, stage of the tumor, PS, metastasis and LFTs were the most commonly adjusted variables in the multivariate analysis. All studies excepting one [4] concluded that higher serum albumin levels were associated with better survival.

Six studies were reviewed on female cancer patients. Four were conducted in ovarian and 2 in breast cancer. All 6 studies were retrospective. The sample size studied ranged from 78 to 1189. Serum albumin was either used as a categorical variable (with 3.5 g/dL as the cut off) or continuous variable. Age, stage of the tumor, BMI, PS, metastasis, treatment history and LFTs were the most commonly adjusted variables in the multivariate analysis. Consistent with studies reviewed under gastrointestinal and lung cancers, lower levels of serum albumin were associated with poor survival in all 6 studies.

Six studies were reviewed on patients with multiple cancer types. Of these, 3 studies were retrospective and 3 prospective. The sample size studied ranged from 76 to 519. The studies used a variety of albumin cut offs, the most commonly used being 3.5 g/dL. Age, primary site, stage of the tumor, BMI, blood counts, metastasis, comorbidities, PS and LFTs were the most commonly adjusted variables in the multivariate analysis. Lower levels of serum albumin were associated with poor survival in all studies.

Finally, we reviewed 8 studies conducted on patients with other cancer sites. Two studies were done on renal cancer patients while one each on head and neck cancer, glioblastoma multiforme NHL, soft tissue sarcoma, Ewing's sarcoma and unknown primaries. Of these 8 studies, 7 were retrospective and 1 was based on a convenience sample. One study used an albumin cut off of 3.85 g/dL [89]. Age, primary site, stage of the tumor, BMI, blood counts, metastasis, treatment regimens, PS and LFTs were the most commonly adjusted variables in the multivariate analysis. Lower levels of serum albumin were found to be associated with poor survival in all studies.

The advantages and disadvantages of serum albumin as an indicator of nutritional status deserve some mention. Serum albumin level is not only a window into the patient's nutritional status but also a useful factor for predicting patient prognosis [63]. Lower levels of serum albumin are indicative of an ongoing systemic response that causes the loss of these proteins [50,67]. The potential advantage of serum albumin level as a pretreatment prognostic factor in cancer patients is that it is inexpensive, reproducible and powerful [50]. When clinical trials are conducted, the success of randomization can be evaluated by comparing pretreatment serum albumin levels in the two arms [91]. Finally, because low levels of serum albumin are associated with poor outcome in cancer patients, perhaps serum albumin can be used as an independent indicator of the need for aggressive nutrition intervention [46]. Among the main disadvantages, the interpretation of serum albumin is often difficult because non-nutritional factors, such as hydration state and disease process, can obscure the effects of actual nutrient deprivation [94]. Furthermore, serum albumin has a relatively long half-life, thus, assessing changes in the nutritional status over a short period of time is challenging [77].

Like most other systematic reviews, this review also suffers from potential publication bias. In general, this bias exists when studies reporting positive associations are more likely to get published. It remains possible that some studies containing valuable data might have gone undetected. Since we restricted this systematic review to include studies published in English only, it is possible that language bias might have affected our conclusions. Despite these limitations, we believe that the extensive available literature reviewed here demonstrates a strong prognostic role of serum albumin in predicting cancer survival. Future studies should evaluate the association between serum albumin levels and patient quality of life. Studies should also prospectively evaluate whether nutritional intervention could have a positive impact on serum albumin levels with a subsequent improvement in patient survival.

In summary, pretreatment serum albumin levels provide useful prognostic significance in cancer. Accordingly, serum albumin level could be used in clinical trials to better define the baseline risk in cancer patients. A critical gap for demonstrating causality, however, is the absence of clinical trials demonstrating that raising albumin levels by means of intravenous infusion or by hyperalimentation decreases the excess risk of mortality in cancer.

## Competing interests

The authors declare that they have no competing interests.

## Authors' contributions

DG and CGL participated in concept, design, data collection, data interpretation and writing. Both authors read and approved the final manuscript.
